# Network connectivity predicts effectiveness of responsive neurostimulation in focal epilepsy

**DOI:** 10.1093/braincomms/fcac104

**Published:** 2022-04-26

**Authors:** Joline M. Fan, Anthony T. Lee, Kiwamu Kudo, Kamalini G. Ranasinghe, Hirofumi Morise, Anne M. Findlay, Heidi E. Kirsch, Edward F. Chang, Srikantan S. Nagarajan, Vikram R. Rao

**Affiliations:** 1 Department of Neurology and Weill Institute for Neurosciences, University of California San Francisco, San Francisco, CA, USA; 2 Department of Neurosurgery, University of California San Francisco, San Francisco, CA, USA; 3 Medical Imaging Center, Ricoh Company, Ltd., Kanazawa, Japan; 4 Department of Radiology and Biomedical Imaging, University of California San Francisco, San Francisco, USA

**Keywords:** RNS system, neuromodulation, imaginary coherence, functional connectivity, magnetoencephalography

## Abstract

Responsive neurostimulation is a promising treatment for drug-resistant focal epilepsy; however, clinical outcomes are highly variable across individuals. The therapeutic mechanism of responsive neurostimulation likely involves modulatory effects on brain networks; however, with no known biomarkers that predict clinical response, patient selection remains empiric. This study aimed to determine whether functional brain connectivity measured non-invasively prior to device implantation predicts clinical response to responsive neurostimulation therapy. Resting-state magnetoencephalography was obtained in 31 participants with subsequent responsive neurostimulation device implantation between 15 August 2014 and 1 October 2020. Functional connectivity was computed across multiple spatial scales (global, hemispheric, and lobar) using pre-implantation magnetoencephalography and normalized to maps of healthy controls. Normalized functional connectivity was investigated as a predictor of clinical response, defined as percent change in self-reported seizure frequency in the most recent year of clinic visits relative to pre-responsive neurostimulation baseline. Area under the receiver operating characteristic curve quantified the performance of functional connectivity in predicting responders (≥50% reduction in seizure frequency) and non-responders (<50%). Leave-one-out cross-validation was furthermore performed to characterize model performance. The relationship between seizure frequency reduction and frequency-specific functional connectivity was further assessed as a continuous measure. Across participants, stimulation was enabled for a median duration of 52.2 (interquartile range, 27.0–62.3) months. Demographics, seizure characteristics, and responsive neurostimulation lead configurations were matched across 22 responders and 9 non-responders. Global functional connectivity in the alpha and beta bands were lower in non-responders as compared with responders (alpha, p_fdr_ < 0.001; beta, p_fdr_ < 0.001). The classification of responsive neurostimulation outcome was improved by combining feature inputs; the best model incorporated four features (i.e. mean and dispersion of alpha and beta bands) and yielded an area under the receiver operating characteristic curve of 0.970 (0.919–1.00). The leave-one-out cross-validation analysis of this four-feature model yielded a sensitivity of 86.3%, specificity of 77.8%, positive predictive value of 90.5%, and negative predictive value of 70%. Global functional connectivity in alpha band correlated with seizure frequency reduction (alpha, *P* = 0.010). Global functional connectivity predicted responder status more strongly, as compared with hemispheric predictors. Lobar functional connectivity was not a predictor. These findings suggest that non-invasive functional connectivity may be a candidate personalized biomarker that has the potential to predict responsive neurostimulation effectiveness and to identify patients most likely to benefit from responsive neurostimulation therapy. Follow-up large-cohort, prospective studies are required to validate this biomarker. These findings furthermore support an emerging view that the therapeutic mechanism of responsive neurostimulation involves network-level effects in the brain.

## Teaser

To prognosticate outcomes with neurostimulation for epilepsy, Fan et al. investigate functional network connectivity measured non-invasively with magnetoencephalography as a novel biomarker for effectiveness of responsive neurostimulation (RNS) therapy. Resting-state functional connectivity in alpha and beta frequency bands predicted response to subsequent RNS therapy and correlated with seizure frequency reduction.

## Graphical Abstract

**Figure:**
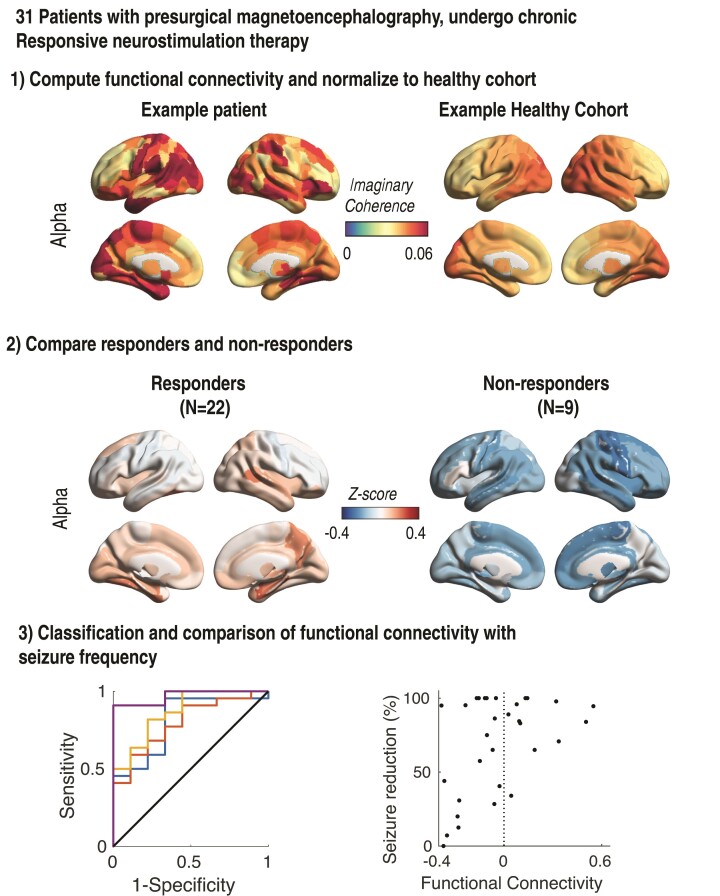
Depicting truncated methodology and findings. Thirty-one patients underwent presurgical magnetoencephalography (MEG) evaluation prior to responsive neurostimulation (RNS) implantation. First, resting-state functional connectivity was computed based on presurgical MEG and normalized to healthy individuals. Second, averaged functional connectivity between RNS responders and non-responders were compared. Third, the classification performance using functional connectivity and the relationship between seizure reduction and functional connectivity were evaluated.

See Hitten Zaveri (https://doi.org/10.1093/braincomms/fcac114) for a scientific commentary on this article.

## Introduction

Of the 46 million people worldwide with active epilepsy,^1^ approximately one-third have seizures that are incompletely controlled with medications. For many of these individuals with drug-resistant epilepsy, surgical resection of seizure-producing brain tissue has the potential to yield seizure freedom.^2^ However, resection may not be feasible in patients with multiple seizure foci or seizures that arise from critical brain regions. In these cases, implanted neurostimulation devices, such as the responsive neurostimulation (RNS®) system, represent promising treatment alternatives. In a recent prospective study, treatment with the RNS system demonstrated progressive clinical benefit with a median reduction in seizure frequency of 75% after nine years.^3–5^

Although median outcomes in clinical trials are encouraging, response to RNS therapy is highly variable across individuals. While over a third of patients experience dramatic improvement with reductions in seizure frequency exceeding 90%, nearly a quarter of patients are non-responders, exhibiting <50% seizure frequency reduction.^3,5^ Owing to a lack of methods to prognosticate even such extreme outcomes, patient selection for RNS in contemporary practice is largely empiric.^6^ Indeed, response does not appear to depend on age at seizure onset, seizure onset zone (SOZ) location, brain imaging abnormalities, or the number of seizure foci.^5^ Similar to all invasive therapies, implantation of the RNS system is associated with morbidity for patients and substantial costs to the medical system.^7^ The desire to minimize ineffective implants creates a critical need for biomarkers that reveal which patients are most and least likely to benefit from RNS therapy.

Recently, electrographic biomarkers of clinical response have been identified in RNS system electrocorticograms^8–10^ and in pre-implant intracranial electroencephalography (iEEG).^11^ These biomarkers derive from invasive recordings and spatially restricted sampling of the epileptogenic network, limiting their clinical utility. An ideal biomarker would be measurable non-invasively, before device implantation, and would not depend on the specific brain regions sampled by intracranial electrodes.

Physiological mechanisms underlying the efficacy of RNS are incompletely understood,^12,13^ however, given the protracted time course of clinical response, they likely involve plasticity and gradual restructuring of the epileptogenic network.^8,14–16^ Growing evidence for network-level effects of chronic neurostimulation^8–10,17^ suggests that intrinsic network connectivity may mediate the effects of neurostimulation and help determine the potential for long-term evolution of the network. As such, we hypothesized that different patterns of network functional connectivity, readily assayed by resting-state magnetoencephalography (MEG), may confer differential susceptibility to chronic neurostimulation and thereby help predict the effectiveness of RNS therapy. To test this hypothesis, we examined a retrospective cohort of patients treated with the RNS system and evaluated their clinical outcomes in relation to resting-state FC measured by MEG prior to device implantation.

## Methods

### Study cohort

All patients (N = 34) who had MEG imaging and were subsequently implanted with the RNS system (NeuroPace, Inc., Mountain View, CA) between 15 August 2014 and 1 October 2020 at UCSF Medical Center were considered for inclusion in this study. Two patients were excluded because they were seizure-free after implantation and RNS stimulation was never enabled. One patient was excluded due to stimulation-related side effects, preventing stimulation from being enabled as intended. The remaining patients (N = 31) were analyzed in this study. In addition, a subgroup analysis was performed on a subset of patients who did not undergo concurrent resective surgery (N = 21). An intention-to-treat analysis was furthermore performed on all patients (N = 34), including the three patients who were excluded in the primary analysis due to stimulation not being enabled or not active on both leads.

Age-matched healthy controls (N = 15) were recruited from the community with eligibility criteria including normal cognition, normal MRI, and absence of neurological or psychiatric illness. Data collection and analysis of MEG and RNS data were approved by the UCSF Institutional Review Board Committee, and all patients provided written consent for the analyses performed in this study.

### Magnetoencephalography data acquisition and preprocessing

All participants in the epilepsy cohort underwent a 1 h, clinically indicated routine EEG/MEG recording in the UCSF Biomagnetic Imaging Laboratory, using a whole-head MEG system (CTF, Port Coquitlam, British Columbia, Canada) comprising 275 axial gradiometers. Fiducial coils over the nasion and bilateral preauricular points were used to align the head position within the sensory array and to co-register MEG data with an individual’s brain MRI. Age-matched controls underwent a shorter MEG recording session lasting 5–10 min. All participants were required to be awake and interactive immediately prior to the recording session. Participants were then instructed to rest quietly in the scanner with eyes closed during the recording. MEG recording sessions were performed while the participants were on their normal antiseizure medications (ASMs); participants who were cognitively altered from their baseline or who had a seizure before or during MEG recording were not included in this study.

Raw EEG/MEG traces were parsed into 15 s epochs. Each segment was directly visualized, and any segments with movement/electrical artifact or epileptiform discharges were removed from the data set. The first six epochs to represent an awake,^18^ artifact-free and epileptiform discharge-free resting state were included in the analysis. The 15 s epochs were concatenated to achieve a 90 s time series representing the resting state. Prior work has demonstrated that 60 s of resting-state data reliably achieves stationarity.^19^ In select patients, a dual signal subspace project filter was used to remove metallic artifact from non-cranial implants.^20^ Source reconstruction was performed using an adaptive beamforming method^21^ to determine the voxel level time series from the sensor time series.

### Magnetoencephalography network functional connectivity

Utilizing the Brainnetome atlas,^22^ the voxel level time series was mapped onto 218 cortical, atlas-based parcellations or regions of interest (ROIs). Time series per ROI were band-pass filtered into the following frequency bands: delta (1–4 Hz), theta (4–8 Hz), alpha (8–14 Hz), beta (14–30 Hz), and low gamma (30–58 Hz). Using the FieldTrip MATLAB Toolbox^23^ and custom built MATLAB tools, FC between ROIs were computed based on imaginary coherence, an established spectral coherence measure that is robust to volume conduction effects.^24^ The 218 cortical ROI level spatial maps were reduced in resolution to 44 modular ROI level maps to increase the signal to noise ratio, giving rise to 990 functional connections, which includes the averaged imaginary coherence within each module. To accommodate for the diverse locations of seizure foci and the native neurophysiologic features of each region, the FC maps of participants were normalized to that of healthy controls by z-scoring the FCs of each modular ROI–ROI connection to the corresponding modular ROI–ROI connections from a healthy cohort (Supplementary Fig. 1, N = 15). The normalization processing enabled the identification of relative increases or decreases in network connectivity compared with a common basis.

FC was computed for global and regional (hemispheric and lobar) spatial representations. Global FC was computed by averaging *z*-scores across all ROI–ROI interactions, spanning the whole brain for each participant. Hemispheric FC was computed as the mean *z*-score within the relevant hemisphere. The relevant hemisphere is determined by the location of the RNS leads, which are placed at the hypothesized SOZ and are thus a marker of the suspected SOZ. If RNS leads were placed bilaterally, the hemispheric FCs were averaged across both hemispheres. Lobar FC was computed as the mean *z*-scores within the lobe containing the RNS lead. If RNS leads involved two lobes, then the FCs per lobe were averaged together for each patient. The relevant lobe and hemisphere for each patient were determined by evaluating post-implantation CT scans and identifying the locations of active leads connected to the device.

### Responsive neurostimulation outcomes

Clinical outcomes were based on averaged patient-reported seizure frequency determined at the most recent clinic visit of two time-samplings performed across all patients between October 2020 through October 2021, to reduce the noise from single time-point measures. Only clinic visits that exceeded a minimum of 6 months after stimulation onset were included. Seizure frequency was quantified relative to their pre-implant baseline (average seizure frequency over the 3 months immediately preceding RNS system implantation). Two patients underwent resective surgery in the years following RNS implantation, due to persistent breakthrough seizures despite RNS therapy. For these two patients, seizure frequency was documented prior to the definitive resection, occurring 63.8 and 39.2 months after stimulation onset. Patient-reported seizure frequency is the most widely used metric to determine clinical response to RNS.^3–5,25–29^ For example, a participant who previously had seven seizures per week and now has three seizures per week is computed to have 57% seizure frequency reduction, i.e. the difference between the current and baseline frequency divided by the baseline frequency. Categorical testing was performed based on the standard definitions^5^ of participants with ≥50% reduction in seizure frequency as ‘responders’ and those with <50% reduction in seizure frequency as ‘non-responders’.

### Statistical analysis

The relationship between RNS outcomes and FC was assessed through both categorical and continuous statistical testing. The distributions of normalized imaginary coherence across all ROI–ROI interactions were obtained for each participant. Whole-brain spatial map visualizations depicted the mean imaginary coherence or *z*-score for each ROI, i.e. the averaged interaction between the represented ROI and all other ROIs. To evaluate group-level statistics of imaginary coherence between responders and non-responders, statistical testing was performed using a linear mixed effects model (RStudio V 1.2.5033). The linear mixed effects model was performed for each frequency band and compared the *z*-scored FCs between responders and non-responders with lobar ROI as a repeated measure. To account for multiple comparisons across frequency bands, we then applied a *post hoc* multiple comparison adjustment (5% false discovery rate, FDR). Individual models were constructed for global and regional FC approaches.

The distributions of ROI–ROI interactions were additionally represented by mean and SD. Receiver operating characteristic (ROC) curves were computed by sweeping through all classification thresholds to elucidate the decision boundary and trade-off between sensitivity and specificity. The area under the ROC curve (AUC) was calculated as a measure of binary classification performance aggregated across all thresholds. Confidence intervals were determined using 1000 bootstrap replicates.^30^ Multivariate logistic regression was used to combine multiple features into a classification model. Specifically, two logistic regression models were constructed to predict responder status using multivariate features of the mean FC of the alpha and beta bands (i.e. 2 features), as well as the mean and SD of alpha and beta FCs (i.e. 4 features). Using R (Rstudio V 1.2.5033), logistic regression models were constructed using the generalized linear model (glm) function with a binary outcome (i.e. setting family-type to binomial). Multivariate features were inputted as linear features for the binary outcome of responder or non-responder. Prediction scores were then obtained from the fitted logistic regression model via the predict.glm function.

To assess further the logistic regression model performance, we performed leave-one-out cross-validation (LOOCV). The LOOCV was performed by splitting the observations into a training and testing set, in which all samples except for a single observation were used in the training set. The logistic regression model was constructed from the training set and tested on the single observation in the testing set. This process was iterated through all samples (e.g. 31 different models built on 30 samples), such that each sample served as the testing set. The accuracy, sensitivity, and specificity were computed. The optimal thresholds for class prediction were determined by maximizing the geometric mean of sensitivity and specificity. In addition, precision-recall curves were constructed based on LOOCV prediction scores and iterating across all classification thresholds to delineate the trade-off between precision and recall.

Finally, a *post hoc* analysis was performed to probe the association between mean/SD FC and RNS outcome as a continuous variable for frequency bands identified in the prior categorical analysis. The association was assessed using the Spearman rank correlation.

### Data Availability

The data that support the findings of this study are available from the corresponding author upon reasonable request.

## Results

### Participant demographics and seizure characteristics

Table 1 demonstrates demographics, seizure characteristics, and RNS lead configurations for all participants (N = 31, 19 females), stratified by responder status. Mean age, duration of epilepsy, etiology, duration with RNS stimulation enabled, and baseline seizure frequency were not significantly different between responders and non-responders. There were no statistical differences in other characteristics, including seizure classification, lobar localization, RNS lead laterality, and prior or concurrent resection at the time of RNS implantation. In addition, there were no statistically significant differences between cohorts in the number or mechanism-of-action of ASMs used (Supplementary Fig. 2, Supplementary Table 1). One participant had bilateral mesial temporal lobe epilepsy. Indications for RNS in the other participants included involvement of eloquent cortex, multiple seizure foci, or regional neocortical epilepsy.^25^ Furthermore, as RNS leads are placed at the hypothesized SOZ, their locations serve as a proxy for the suspected SOZ. Demographics of the healthy controls (N = 15) were age and gender matched to the epilepsy cohort (Supplementary Table 2).

**Table 1:** Participant characteristics, stratified by responder (R) and non-responder (NR) status

	All participants (N = 31)	R (N = 22)	NR (N = 9)	*P*-values^a^
Age, y	32.0 (24.3–39.0)	33.5 (25.0–39.0)	27.0 (23.0–40.0)	0.349
Gender, F (%)	19 (61.3)	14 (63.6)	5 (55.6)	0.704
Duration of epilepsy, y	14.0 (10.0–23.5)	14.0 (10.0–21.0)	17.0 (13.8–26.8)	0.198
Duration stimulation enabled, mos	52.2 (27.0–62.3)	52.8 (41.3–62.0)	50.9 (15.3–66.3)	0.948
Number of ASMs, no.	2.42 (0.76)	2.36 (0.79)	2.56 (0.73)	0.671
Etiology, no. (%)				0.508
Cryptogenic	16 (51.6)	11 (50)	5 (55.6)	—
Encephalitis	2 (6.5)	1 (4.5)	1 (11.1)	—
AVM	2 (6.5)	1 (4.5)	1 (11.1)	—
PVNH	5 (16.1)	4 (18.2)	1 (11.1)	—
Genetic/developmental	2 (6.5)	2 (9.1)	0 (0)	—
FCD	3 (9.7)	3 (13.6)	0 (0)	—
Stroke	1 (3.2)	0 (0)	1 (11.1)	—
Seizure type^b^, no. (%)				0.079
FAS	16 (51.6)	15 (68.2)	1 (11.1)	—
FIAS	22 (71.0)	14 (63.6)	8 (88.9)	—
FBTC	15 (48.4)	10 (45.5)	5 (55.6)	—
Baseline seizure frequency, per wk	3.5 (1.0–9.3)	6.0 (2.0–14.0)	1.0 (0.4–4.4)	0.070
RNS lead locations^c^, no. (%)				0.233
Frontal	17 (54.8)	10 (45.4)	7 (77.8)	—
Neocortical temporal	20 (64.5)	15 (68.2)	5 (55.6)	—
Mesial temporal	9 (29.0)	8 (36.4)	1 (11.1)	—
Insular	2 (6.5)	0 (0)	2 (22.2)	—
Parietal	11 (35.5)	8 (36.4)	3 (33.3)	—
Occipital	2 (6.5)	2 (9.1)	0 (0)	—
Other	1 (3.2)	1 (4.5)	0 (0)	—
Prior resection, Y (%)	8 (25.8)	7 (31.8)	1 (11.1)	0.379
Concurrent resection, Y (%)	10 (32.3)	8 (36.4)	2 (22.2)	0.677
RNS lead types, no. (%)				0.569
Strips only	23 (74.2)	15 (68.2)	8 (88.9)	—
Depths only	1 (3.2)	1 (4.5)	0 (0)	—
Neocortical + Depth	7 (22.6)	6 (27.3)	1 (11.1)	—
RNS lead lateralization, no. (%)				0.459
Right	6 (19.3)	3 (13.6)	3 (33.3)	—
Left	22 (71.0)	17 (77.3)	5 (55.6)	—
Both	3 (9.7)	2 (9.1)	1 (11.1)	—

Values for age, duration of epilepsy, duration stimulation enabled, and baseline seizure frequency are given in medians with interquartile ranges in parentheses. Values for number of ASMs are given in means with standard deviations in parentheses.

^a^ Differences between R (≥50% seizure reduction) and NR (<50% seizure reduction). Statistical testing performed by the Wilcoxon–Mann–Whitney test for two-sample comparisons. Fisher’s exact testing was performed for categorical testing; *post hoc P*-values from multiple comparison testing is provided if Fisher’s exact testing met significance, a = 0.05.

^b^ May include more than one type for individual participants.

^c^ Counts include each lead per participant.

ASM = antiseizure medications; AVM = arteriovenous malformation; PVNH = periventricular nodular heterotopia; FCD = focal cortical dysplasia; FAS = focal aware seizure; FIAS = focal impaired awareness seizure; FBTC = focal to bilateral tonic–clonic seizure.

### Spatial functional connectivity maps for responders and non-responders

**Figure 1:**
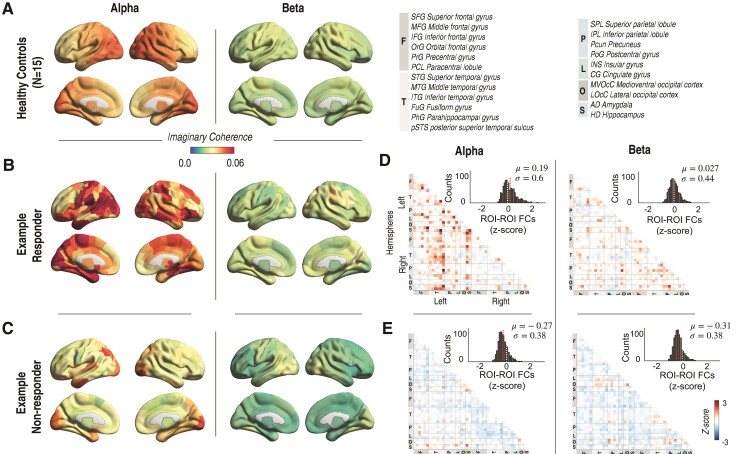
**Representative global and region-to-region FC maps in the alpha and beta band for a responder and non-responder.** (**A**). Global FC spatial maps of healthy controls (averaged across N = 15) for the alpha (*left*) and beta (*right*) frequency bands. (**B**). Global FC spatial maps for an example responder, revealing regions of elevated FC in the alpha band (*left)*. (**C**). Global FC spatial map for an example non-responder, revealing regions of reduced FC in both the alpha (*left*) and beta (*right*) frequency bands. (**D**). Normalized region-to-region FC map for the example responder in the alpha (*left*) and beta (*right*) bands. Normalization involves *z*-scoring a participant’s FC map to the region-to-region FC maps of the healthy controls. Inset demonstrates the distribution of normalized FCs for the representative responder in the alpha and beta bands with global mean (SD) of 0.19 (0.6) and 0.027 (0.44), respectively. The red dotted line indicates the mean of the normalized FC distribution. The white dotted line indicates the null hypothesis for all FCs, i.e. the healthy control. (**E**). Normalized region-to-region FC map for the non-responder, revealing low region-to-region FCs, as compared to healthy individuals. Inset reveals the distribution of normalized FCs for the representative non-responder in the alpha and beta bands with global mean (SD) of −0.27 (0.38) and −0.31 (0.38), respectively.

To quantify intrinsic network connectivity, spatial FC maps were computed for each participant using pre-implant MEG. Fig. 1A–C compare spatial maps of a representative responder and non-responder to spatial FC maps averaged across healthy controls. Relative to controls, elevated FC is observed in the alpha band of the example responder, whereas reduced FC is observed in alpha and beta bands of the example non-responder. Region-to-region FCs of participants were normalized to those of healthy controls to facilitate comparison across participants with diverse epileptogenic networks. Normalized region-to-region FC maps demonstrate elevated connectivity in the alpha band of the responder and reduced connectivity in the alpha and beta bands of the non-responder (Fig. 1D and E). Summary statistics of the distribution of normalized region-to-region FCs in alpha and beta bands demonstrate an increased mean and dispersion (standard deviation, SD) in the responder, as compared with the non-responder (Fig. 1D and E, inset).

### Functional connectivity as a predictor of responder and non-responders

**Figure 2:**
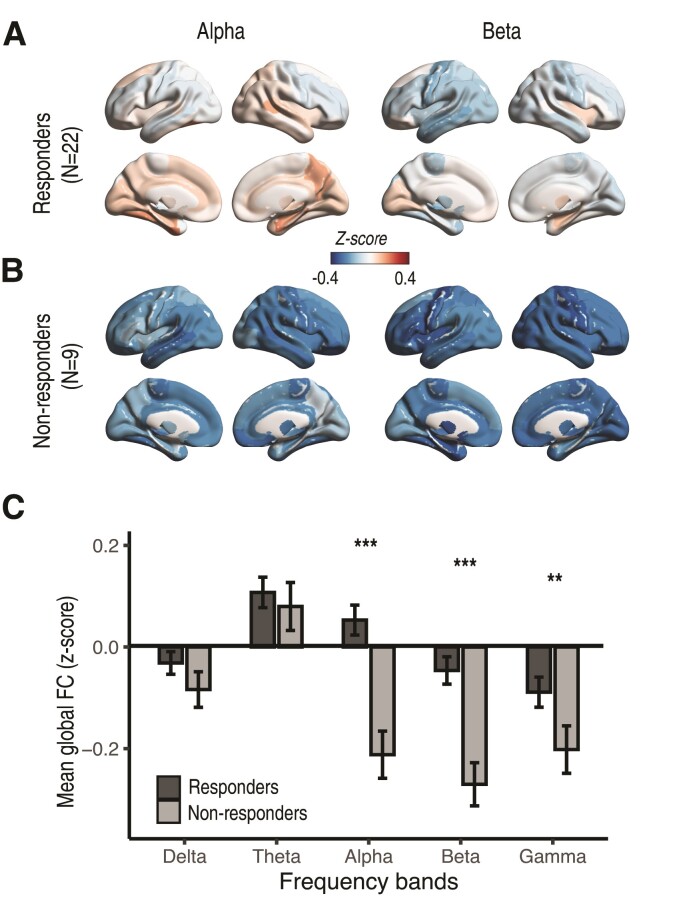
**Group analysis revealing frequency-specific patterns of global FC in the responder and non-responder cohorts.** (**A**). Group-averaged spatial maps of responders reveal global and regional increases in FC in the alpha band (*left*) and reduced FC in the beta band (*right*), relative to healthy cohorts. *Z*-scores for each patient and each ROI are computed relative to the healthy cohort. (**B**). Group-averaged spatial maps of non-responders demonstrate broadly reduced FC in both the alpha (*left*) and beta (*right*) bands, relative to healthy cohorts and responders. (**C**). Mean global FCs, averaged across the spatial maps, are increased in responders as compared to non-responders in alpha, beta, and gamma bands (alpha, p_fdr_ < 0.001; beta, p_fdr_ < 0.001; gamma, p_fdr_ = 0.004; *P*-values adjusted with 5% FDR). Positive and negative values indicate increased and decreased connectivity relative to healthy individuals, respectively. Statistical testing for each frequency band is obtained from a linear mixed effects model comparing the *z*-scored FCs between responders and non-responders with lobar ROI as a repeated measure. P-values are corrected via a *post hoc* multiple comparison correction across frequency bands (FDR level 0.05). LS-means and 95% confidence limits from the linear mixed effects model are depicted in C. Number of asterisks indicates significance values of p_fdr_ < 0.05, p_fdr_ < 0.01, and p_fdr_ < 0.001, respectively.

Group-averaged spatial maps of normalized FCs demonstrated global network changes with responders exhibiting higher FC in alpha and beta bands as compared to non-responders (Fig. 2A and B). Averaging the FCs across the global spatial maps, responders were found to have higher mean FC in the alpha, beta, and gamma bands, as compared with non-responders (Fig. 2C, alpha, p_fdr_ < 0.001; beta, p_fdr_ < 0.001; gamma, p_fdr_ = 0.004). Responders had positive mean normalized FC in the alpha band, implying an increase in mean connectivity relative to healthy controls. In contrast, non-responders had negative mean normalized FCs in the alpha frequencies, suggesting an overall reduction in connectivity as compared to the healthy controls. Both responders and non-responders had negative mean normalized FC in the beta and gamma frequencies, but non-responders exhibited higher magnitudes, implying more severely disrupted connectivity.

Regional FC demonstrated less robust frequency-specific differentiation between responders and non-responders than global FC. Hemispheric FC did not yield statistical differences between responders and non-responders in the gamma band (p_fdr_ = 0.067) but continued to demonstrate a statistically significant increase in the alpha and beta bands in responders as compared with non-responders (alpha, p_fdr_ < 0.001; beta, p_fdr_ < 0.001). Although revealing similar trends, the lobar FC did not yield significant findings in any frequency band.

**Figure 3:**
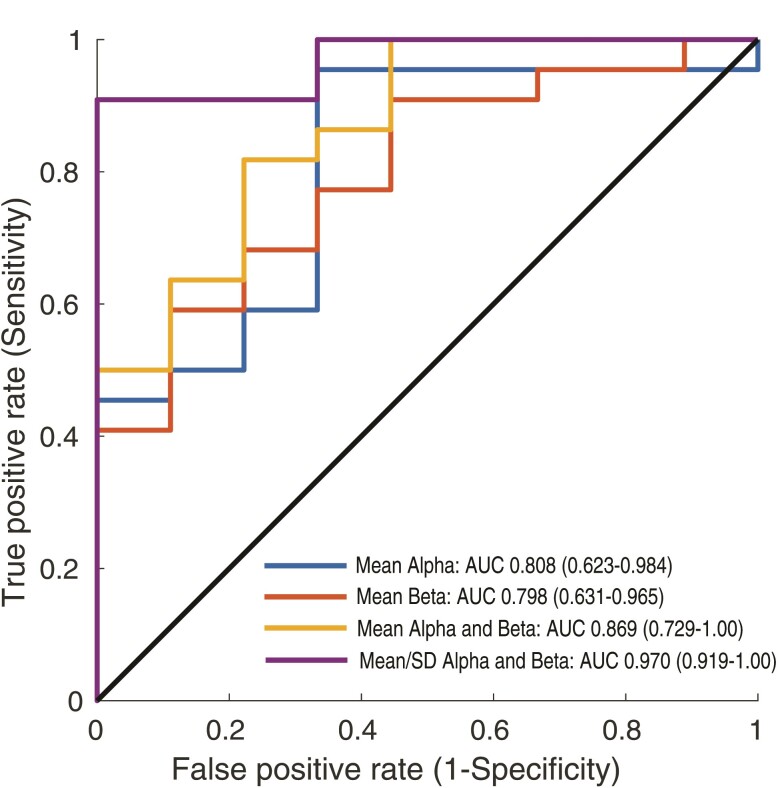
**Global FC predicts RNS response. ROC curves for classification of responders and non-responders using frequency-specific global FC.** Predictors include the mean global FC within the alpha (blue) and beta (red) frequency bands. In addition, two logistic regression models combining mean FC of the alpha/beta frequency bands (yellow) and the mean/SD of the alpha/beta frequency bands (purple) are demonstrated. AUC is highest in the logistic regression model that combines both the mean and SD within the alpha and beta frequency bands (AUC: 0.970, 95% CI: 0.919–1.000).

Given their prominent and robust differences between responders and non-responders (Fig. 2C), alpha and beta frequency bands were used in subsequent classification models. ROC curves were constructed to evaluate the feasibility of classifying responders versus non-responders using the mean global FC (Fig. 3), yielding AUCs of 0.808 (95% CI: 0.632–0.984) and 0.798 (95% CI: 0.631–0.965) for alpha and beta frequency bands, respectively. A combined logistic regression model using the alpha and beta frequency bands yielded an AUC of 0.869 (95% CI: 0.729–1.000). Dispersion of FCs (SD) was further assessed as a predictor of responder status, to capture a different dimension of the distribution of connectivity strengths. In a corresponding ROC curve analysis, the SD of FCs yielded AUCs of 0.884 (95% CI: 0.760–1.000) and 0.783 (95% CI, 0.615–0.951) for alpha and beta frequency bands, respectively. A combined logistic regression model using both the mean and SD of FCs within the alpha and beta frequency bands yielded an AUC of 0.970 (95% CI: 0.919–1.000). The addition of gamma features did not further improve the model. To assess model performance, LOOCV was performed on the best logistic regression model (i.e. combined logistic regression model using four features, mean/SD of FCs within the alpha and beta bands), which yielded an accuracy of 83.9%, sensitivity of 86.3%, and specificity of 77.8%. LOOCV was additionally performed to assess the robustness of the AUC metric (see Supplementary Methods). Finally, precision-recall curves were constructed to further assess model performance (Supplementary Fig. 3). The precision (i.e. positive predictive value, PPV) and negative predictive value (NPV) for the four-feature model were 90.5% and 70.0%, respectively, based on the optimal threshold previously determined by the maximal geometric mean of the specificity and sensitivity. LOOCV metrics for all other features models are additionally presented in Supplementary Table 3.

In a subgroup analysis of patients who did not undergo resection (Supplementary Fig. 4, N = 21, comprised 14 R and 7 NR), the mean global FC in the alpha, beta, and gamma bands remained statistically significant between responders and non-responders (Supplementary Fig. 4, alpha, p_fdr_ < 0.001; beta, p_fdr_ < 0.001; gamma, p_fdr_ < 0.001). In addition, an intention-to-treat analysis was performed, which includes the three patients for whom stimulation was never enabled or was active in only a single lead (N = 34, comprised 25 R and 9 NR). Mean global FC in the alpha, beta, and gamma bands remained statistically significant between responders and non-responders in the intention-to-treat analysis (Supplementary Figure 5, alpha, p_fdr_ < 0.001; beta, p_fdr_ < 0.001; gamma, p_fdr_ = 0.004).

### Association between functional connectivity and seizure frequency reduction

**Figure 4:**
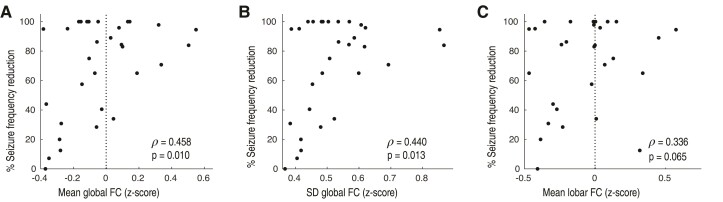
**Alpha band FC predicts degree of seizure frequency reduction.** (**A**) Mean and (**B**) dispersion (SD) of the distribution of region-to-region FC in the alpha band correlates with degree of seizure frequency reduction (mean, ρ = 0.458, *P* = 0.010; SD, ρ = 0.440, *P* = 0.013). (**C**) The association between seizure frequency reduction and lobar FC is not statistically significant (ρ = 0.336, *P* = 0.065).

We next investigated whether frequency-specific FC could provide insight on the degree of seizure frequency reduction, beyond the binary outcome classification of responder versus non-responder. We investigated the two frequency bands, alpha and beta, that most robustly stratified responders and non-responders in the previous analyses. The association between mean normalized FC within the alpha frequency band and seizure reduction revealed the presence of a dose–response relationship (Fig. 4A, Spearman’s correlation: ρ = 0.458, *P* = 0.010). The SD of the FC distribution in the alpha band was also positively correlated with seizure reduction (Fig. 4B, ρ = 0.440, *P* = 0.013). In addition, a positive but reduced correlation was observed for mean hemispheric FC (ρ = 0.417, *P* = 0.019). Mean lobar FC in the alpha band was not significant (Fig. 4C, ρ = 0.336, *P* = 0.065). The correlations of global and regional FC to seizure reduction within the beta band were not significant. In the subgroup analysis of patients who did not undergo resection, the association between seizure reduction and mean global FC in the alpha band remained statistically significant (Supplementary Fig. 4, ρ = 0.562, *P* = 0.008). In addition, the association between seizure reduction and mean global FC in the alpha band remained statistically significant in the intention-to-treat cohort (Supplementary Fig. 5, ρ = 0.409, *P* = 0.016).

## Discussion

Clinical response to RNS therapy is highly variable across patients, and currently, there are no established methods to prognosticate treatment response prior to device implant.^3,5^ Here, using FC maps derived from pre-implant MEG, we demonstrate evidence that elevated global/regional network connectivity is associated with favorable outcomes and reduced global/regional network connectivity is associated with poor outcomes following subsequent treatment with RNS. Although FC in both alpha and beta frequency bands individually demonstrate discriminability between responders and non-responders, the combination of the two bands yields greater predictive value. To our knowledge, frequency-specific FC is the first non-invasive biomarker that has been demonstrated to have potential to predict clinical response to RNS therapy.

Although the therapeutic mechanism of RNS is unknown, current evidence suggests a role for chronic restructuring of the epileptogenic network.^10,14–16^ For example, neurophysiological features, such as the frequency modulation of ictal activity or interictal spike rates, evolve with chronic stimulation.^8,9^ A network-level mechanism presumably underlies the efficacy of RNS for treating spatially-distributed (‘regional’) SOZs, despite the initial conception of RNS as being most well-suited for the treatment of discrete, highly localized SOZs.^25^ Importantly, resting-state FC has been identified to be disrupted in epilepsy cohorts as compared to healthy controls, while subnetworks within the SOZ have been reported to have elevated FC.^31–33^ Indeed, in both the responders and non-responders, FC within the beta band was reduced relative to healthy controls; however, we demonstrate that within the beta band, the non-responders had significantly lower FC as compared with the responder cohort, which remained more intact. In the alpha band, RNS responders exhibited increased FC relative to healthy cohorts, whereas non-responders exhibited decreased connectivity. The elevated FC in specific frequency bands (i.e. alpha/theta in responders and theta in non-responders) may relate to spatial characteristics of the epileptogenic network. Specifically, RNS is often indicated for multifocal or regional SOZs,^27^ and, given that frequency-specific FC can be elevated within the SOZ,^26^ the increased global FC seen here in certain frequency bands may relate to the overall larger contribution of a spatially extensive SOZ to the mean global FC. Prior studies have revealed that FC captures evoked functional networks, such as those engaged by neurostimulation.^34^ A purely speculative possibility is that electrical pulses delivered by the RNS device can more readily diffuse through brain networks with relatively higher global FC, which may in some way potentiate the antiseizure effects of this therapy. Conversely, networks with low global FC may have limited spread of the therapeutic effects of neurostimulation. These findings are consistent with iEEG data suggesting that decreased synchronizability at seizure onset is correlated with poor RNS outcome.^11^ In contrast, increased *local* FC within the resection site^31,35^ or decreased network synchronizability^36^ have been shown to predict favorable surgical resection outcomes, suggesting that brain network characteristics which portend favorable response to neurostimulation versus resection may be as distinct as the therapies themselves.

A major challenge in outcomes prediction of extra-temporal lobe epilepsies involves the diversity of seizure onset locations and the unique neurophysiological features of each location. In this study, we accounted for this heterogeneity by normalizing FC maps of study participants to age-matched healthy controls, which facilitates comparison across patients. Thus, brain regions that naturally have higher FC, such as occipital regions in the alpha frequency band during wakefulness, are accounted for through this normalization process. As such, each patient undergoes the same diagnostic testing, in which MEG sensor data are uniform or ‘templated’ across all patients, and the heterogenous SOZs are accounted for by the normalization process. By contrast, FC biomarkers based on iEEG^11^ are challenged by the disparate and ‘tailored’ electrode locations across patients, oversampling of the SOZ, and the long recording durations often needed to capture seizures.

An additional benefit of utilizing a templated rather than tailored diagnostic approach is that consistent, whole-brain coverage is obtained. Despite its superior temporal resolution, iEEG is tailored for each patient and spatially limited to areas of electrode coverage surrounding the hypothesized SOZ. As network connectivity has been observed to be highest within the epileptogenic core,^31,37^ spatially restricting analysis to the presumed epileptogenic core, i.e. the region adjacent to the RNS leads, could conceivably yield a more robust difference between responders and non-responders. However, in this study, global metrics were more robust than regional measures. With both categorical and continuous testing, we found that spatially restricting the FC analysis from global to regional measures resulted in less discriminability of clinical outcomes. The frequency-specific FC trends between responders and non-responders were similar between global and regional FC but less robust in hemispheric measures and absent in lobar measures. In addition, the correlation between FC and seizure reduction decreased from global to regional measures. Because the epileptogenic network is known to be distributed broadly,^38–41^ these findings suggest that whole-brain coverage may capture critical network features, including regions that are not directly adjacent to the hypothesized SOZ.^42^

Outside of the epileptogenic network, large-scale network impairments as measured by reduced FC have also been observed in people with epilepsy.^43–45^ Consistent with prior findings, responders and non-responders were both observed to have regions of reduced FC. We demonstrate that the mean and SD of the FC distribution independently predict RNS response, and that including both summary statistics improves performance of the classifier. One interpretation of this finding is that overall network connectivity and the heterogeneity of regional connectivity are both salient determinants of response to RNS therapy. This is consistent with recent studies showing that network topology influences the effects of neurostimulation.^46,47^ Network biomarkers that probe second-order metrics of the distribution of FCs and intrinsic network topology,^48,49^ such as graph theoretic measures, may also help predict clinical outcome and are of great interest for future work.

Our cohort included participants who were treated with RNS and concurrent partial resection of epileptogenic brain tissue. We elected to keep these participants in the original cohort so as to increase the applicability of this study to real-world settings, where combining RNS and resection is an emerging treatment approach.^50^ As these participants were distributed equally across both the responder and non-responder cohorts (Table 1), their inclusion is unlikely to drive our results. Furthermore, subgroup analysis confirmed that our central findings held even when patients treated with resection were excluded (Supplementary Fig 4).

Limitations of this study include the modest sample size and challenges inherent within clinically relevant, patient-reported seizure frequency. While RNS remains an emerging therapy, the sample size in this study is further constrained by the clinical selection of patients who typically undergo diagnostic presurgical MEG studies. In addition, the asymmetric proportion of responders and non-responders in this study reflects the distribution of RNS outcomes observed in retrospective analyses using a standard definition of ‘responder’ (≥50% seizure frequency reduction).^5^ Future studies with larger sample sizes and cross-validation using hold out validation approaches are necessary to establish the generalizability and error rates of this biomarker for predicting response to RNS. Future prospective studies will also enable a more representative distribution of epilepsies, including a higher proportion of bitemporal epilepsy. In addition, RNS outcomes in this study are based on patient-reported seizure frequency, which is well-known to be prone to recall bias and other sources of error^51^ yet remains the gold standard endpoint in most epilepsy trials. In this work, we attempted to mitigate the errors of single time-point outcome evaluations by averaging the seizure frequency from two fixed assessments in the most recent year. The heterogeneity of the patient cohort is also a limitation of this work. We attempted to control for potential confounders by demonstrating a similar distribution of patient characteristics (e.g. medications, seizure type, etiology; see Table 1) between the two cohorts; however, larger, multicenter studies will be necessary in the future. Other limitations of the retrospective nature of our analysis, include non-standardized RNS stimulation parameters, ASMs, and behavioral factors.

In contemporary practice, some patients who appear to be good candidates for RNS may ultimately respond poorly to this therapy. Without methods to anticipate this outcome, patients must endure device implantation and potentially years of ineffectual device optimization before moving on to other therapies. Here, we demonstrate evidence that frequency-specific global and regional network connectivity may be associated with RNS outcomes and may therefore potentially serve as a personalized biomarker of treatment response. Such a biomarker obtained non-invasively prior to device implantation opens the door to the rational selection of patients who are most likely to benefit from RNS and, potentially, other neurostimulation devices.

## Supplementary Material

fcac104_Supplementary_Data

## Abbreviations

ASM =: antiseizure medication
AUC =: area under the receiver operating characteristic curve
FC =: functional connectivity
FDR =: false discovery rate
iEEG =: intracranial electroencephalography
IQR =: interquartile range
LOOCV =: leave-one-out cross-validation
MEG =: magnetoencephalography
NPV =: negative predictive value
NR =: non-responder
PPV =: positive predictive value
R =: responder
ROC =: receiver operating characteristic
ROI =: region of interest
RNS =: responsive neurostimulation
SD =: standard deviation
SOZ =: seizure onset zone
