# Supplementary material for: Network connectivity predicts effectiveness of responsive neurostimulation in focal epilepsy
Source: Brain Commun. 2022 Apr 26;4(3):fcac104. doi: 10.1093/braincomms/fcac104 (PMC9123848; doi:10.1093/braincomms/fcac104)
Supplement: fcac104_Supplementary_Data [file fcac104_supplementary_data.docx]

**Supplementary Materials**

| **Supplementary Table 1. ASM characteristics across two patient cohorts** | | | |
| --- | --- | --- | --- |
|  | **R (N=22)** | **NR (N=9)** | **P-value*** |
| **CBZ/OXC/ESL** | 12 | 3 | 0.454 |
| **PHT** | 4 | 2 |  |
| **LTG** | 4 | 4 |  |
| **LEV/BRV** | 8 | 6 |  |
| **LCM** | 7 | 4 |  |
| **CLB/CLN/CZP** | 7 | 1 |  |
| **TPM/ZNS** | 8 | 2 |  |
| **PER** | 2 | 0 |  |
| **GBP** | 0 | 1 |  |
|  |  |  |  |
| **Total ASMs** | **52** | **23** | **75** |

*P-value determined based on a categorical Fisher test.

Abbreviations. CBZ = Carbamazepine; OXC = Oxcarbazepine; ESL = Eslicarbazepine; PHT = Phenytoin; LTG = Lamotrigine; LEV = Levetiracetam; BRV = Brivaracetam; LCM = Lacosamide; CLB = Clobazam; CLN = Clonazepam; CZP = Clorazepate; TPM = Topiramate; ZNS = Zonisamide; PER = Perampanel; GBP = Gabapentin

| **Supplementary Table 2. Patient characteristics, Controls vs. Epilepsy** | | | | |
| --- | --- | --- | --- | --- |
|  | **Controls (N=15)** |  | **Epilepsy (N=31)** | **p values** |
| **Age, y** | 31.0 (30.3 - 59.0) | 32.0 (24.3 - 39.0) | | 0.201 |
| **Gender, F (%)** | 7 (46.7) | 19 (61.3) | | 0.527 |

Values for Age are medians with interquartile ranges in parentheses.

^*^Statistical testing performed by the Wilcoxon-Mann-Whitney test for two-sample comparisons of Age. Fisher’s exact testing was performed for categorical testing for gender.

| **Supplementary Table 3. LOOCV metrics corresponding to logistic regression with different feature inputs** | | | | |
| --- | --- | --- | --- | --- |
| **Features** | **Sensitivity** | **Specificity** | **Precision (PPV)** | **NPV** |
| Alpha (mean) | 90.9% | 66.7% | 87.0% | 75.0% |
| Beta (mean) | 90.9% | 55.6% | 83.3% | 71.4% |
| Alpha and Beta (means) | 90.9% | 55.6% | 83.3% | 71.4% |
| Alpha (std) | 81.8% | 77.8% | 90.0% | 63.6% |
| Beta (std) | 81.8% | 55.6% | 81.8% | 55.6% |
| Alpha and Beta (std) | 77.3% | 100.0% | 100.0% | 64.4% |
| Alpha and Beta (means and std) | 86.3% | 77.8% | 90.5% | 70.0% |
| Optimal thresholds were determined to achieve a balance between sensitivity and specificity, calculated by the geometric mean of sensitivity and specificity. PPV and NPV are calculated based on the same optimal threshold.  Abbreviations: PPV = Positive predictive value; NPV = Negative predictive value | | | | |

**
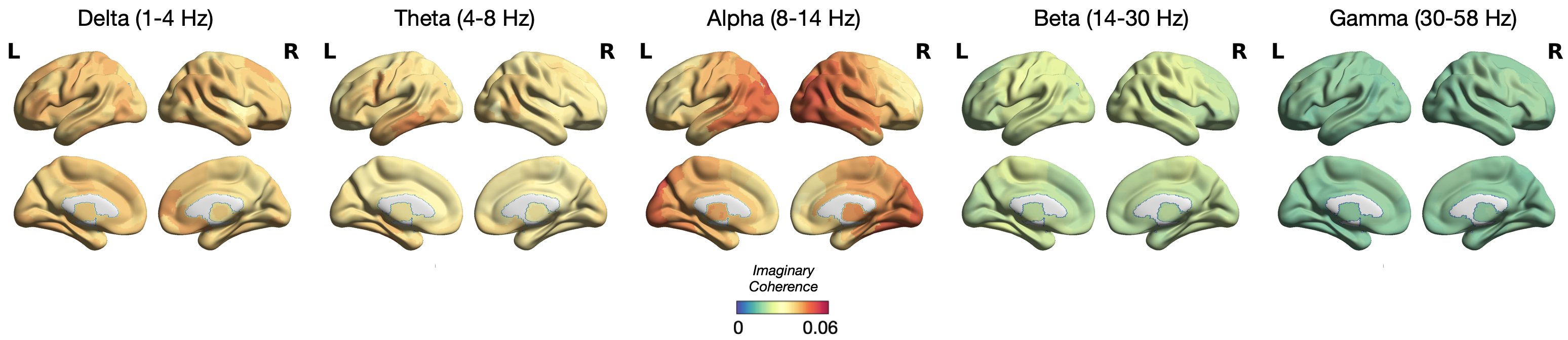
**

**Supplementary Figure 1. Frequency specific global FC maps in healthy controls used to derive subject-specific z-scores.** Healthy cohort source reconstruction maps of FC for individual frequency bands (averaged across N=15 individuals). The healthy control in Fig. 1a is identical to the alpha frequency demonstrated here.

**Supplementary Figure 2: Comparison of the number of anti-seizure medications (ASMs) between the responder (blue) and non-responder (orange) cohort.** There is a no statistical difference in the distribution of number of ASMs between the two cohorts (p=0.671, Mann-Whitney U test).

**Supplementary Figure 3. Precision-recall curves of individual model features and combination of model features**. Precision-recall curves demonstrate the trade-off between precision (i.e. PPV) and recall (i.e. sensitivity) and are based on the LOOCV prediction scores and sweeping across all classification thresholds. The curves are color coded by the different model features, including individual features and combined features.

**Supplementary Figure 4. Global FC and RNS efficacy in a reduced cohort (N=21), excluding participants who did underwent concurrent resective surgery. a.** Frequency specific global FC of R and NR, demonstrating a statistically significant difference in the alpha (p_fdr_ < 0.001), beta (p_fdr_ < 0.001), and gamma (p_fdr_ < 0.001) frequency bands between R and NR. **b.** Mean global FC correlated with degree of seizure reduction in the alpha frequency band (p=0.008).

**
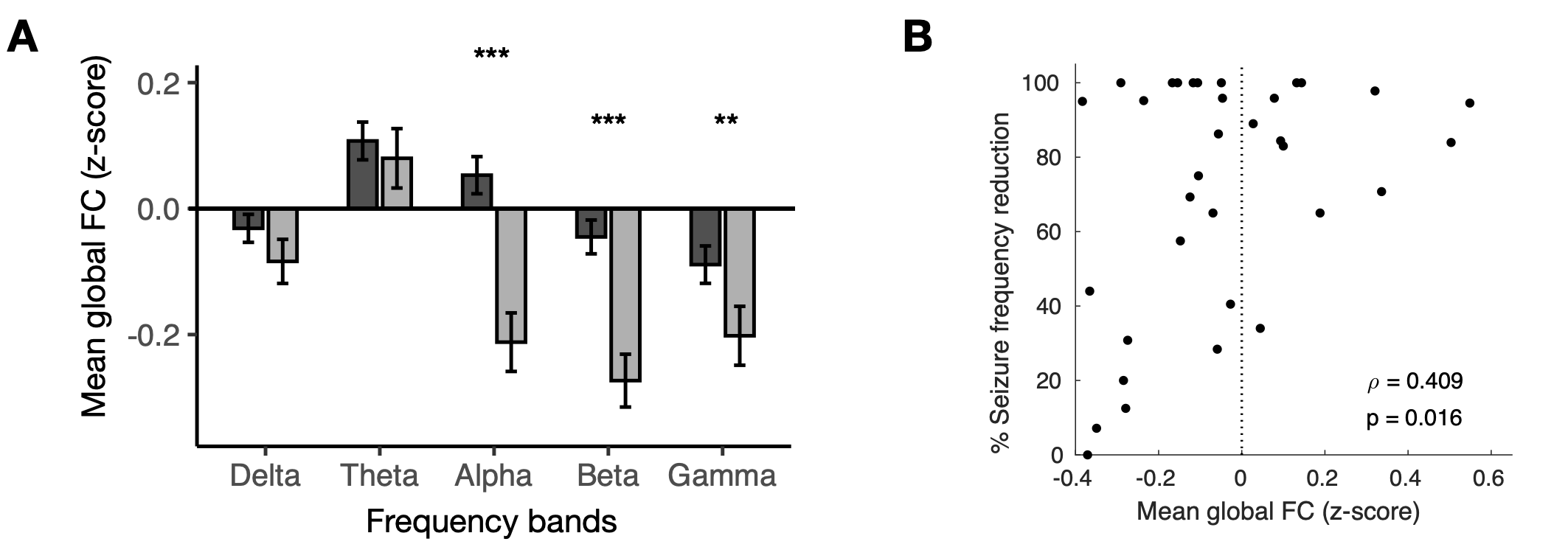
**

**Supplementary Figure 5. Global FC and RNS efficacy in an intention to treat cohort (N=34), which includes patients from the original cohort and patients for whom stimulation was not enabled or not enabled as intended. a.** Frequency specific global FC of R and NR, demonstrating a statistically significant difference in the alpha (p_fdr_ < 0.001), beta (p_fdr_ < 0.001), and gamma (p_fdr_ = 0.004) frequency bands between R and NR. **b.** Mean global FC correlated with degree of seizure reduction in the alpha frequency band (p=0.016).

**Supplementary Methods:**

**Leave-one-out cross-validation (LOOCV) on AUC curve robustness**

In addition to the calculation of model performance to arrive at accuracy, sensitivity, and specificity, we additionally evaluated the robustness of the AUC curve using LOOCV. We computed sequential logistic regression models using the 4-feature model (e.g. 31 different models built on 30 samples) and computed the AUC with respect to a single sample left out. The mean (SD) AUC metric was 0.871 (0.013).
